# Epstein-Barr Virus (EBV) detection and typing by PCR: a contribution to diagnostic screening of EBV-positive Burkitt's lymphoma

**DOI:** 10.1186/1746-1596-1-17

**Published:** 2006-08-07

**Authors:** Rocío Hassan, Lídia Roxana White, Claudio Gustavo Stefanoff, Deilson Elgui de Oliveira, Fabricio E Felisbino, Claudete Esteves Klumb, Carlos E Bacchi, Héctor N Seuánez, Ilana R Zalcberg

**Affiliations:** 1 Bone Marrow Transplantation Center (CEMO), Instituto Nacional de Câncer (INCA), Praça Cruz Vermelha 23, 20230-130, 6^th ^floor, Rio de Janeiro, RJ, Brazil; 2 Department of Pathology, Botucatu School of Medicine, Universidade Estadual Paulista, São Paulo, SP, Brazil; 3 Hematology Service, Instituto Nacional de Câncer (INCA), Praça Cruz Vermelha 23, 20230-130, Rio de Janeiro, RJ, Brazil; 4 Genetics Division, Instituto Nacional de Câncer (INCA), Rua André Cavalcanti 37, 4^th ^floor, 20231-050, Rio de Janeiro, RJ, Brazil

## Abstract

**Background:**

Epstein-Barr virus (EBV) is associated to the etio-pathogenesis of an increasing number of tumors. Detection of EBV in pathology samples is relevant since its high prevalence in some cancers makes the virus a promising target of specific therapies. RNA *in situ *hybridization (RISH) is the standard diagnostic procedure, while polymerase chain reaction (PCR)-based methods are used for strain (EBV type-1 or 2) distinction. We performed a systematic comparison between RISH and PCR for EBV detection, in a group of childhood B-cell Non-Hodgkin lymphomas (NHL), aiming to validate PCR as a first, rapid method for the diagnosis of EBV-associated B-cell NHL.

**Methods:**

EBV infection was investigated in formalin fixed paraffin-embedded tumor samples of 41 children with B-cell NHL, including 35 Burkitt's lymphoma (BL), from Rio de Janeiro, Brazil, by *in situ *hybridization of EBV-encoded small RNA (EBER-RISH) and PCR assays based on EBNA2 amplification.

**Results:**

EBV genomes were detected in 68% of all NHL. Type 1 and 2 accounted for 80% and 20% of EBV infection, respectively. PCR and RISH were highly concordant (95%), as well as single- and nested-PCR results, allowing the use of a single PCR round for diagnostic purposes. PCR assays showed a sensitivity and specificity of 96% and 100%, respectively, with a detection level of 1 EBV genome in 5,000–10,000 EBV-negative cells, excluding the possibility of detecting low-number EBV-bearing memory cells.

**Conclusion:**

We describe adequate PCR conditions with similar sensitivity and reliability to RISH, to be used for EBV diagnostic screening in high grade B-NHL, in "at risk" geographic regions.

## Background

Epstein-Barr (EBV) is a widespread human herpesvirus mainly B-cell tropic but capable of infecting T-cells and epithelial cells [1,2]. Initial exposure to EBV usually occurs in the first decade of life producing persistent, latent asymptomatic infection. EBV infects more than 90% of the healthy population and is maintained at low copy numbers (1–50 × 10^-6 ^cells) in memory B-cells [3,4].

EBV has been associated to the etio-pathogenesis of an increasing number of cancers [1,2,5,6]. In developing countries, prevalence of EBV may reach 80% in some neoplasms, thus, exploitation of EBV association for clinical purposes and therapeutic interventions is of interest [7-10].

Specific sensitive methods for detecting EBV infection are based on *in situ *hybridization (ISH), Southern blotting and PCR [11,12]. RNA-ISH (RISH) for detecting EBERs (EBV transcripts highly expressed in latently infected cells) is the standard procedure for EBV diagnosis allowing identification and distinction of infected cell types [13,14]. PCR-based methods are used for strain determination (type-1 or 2). However, when strictly standardized, PCR may have an important role in EBV diagnosis and management in high-grade non-Hodgkin lymphoma (NHL), although systematic comparisons between RISH and PCR approaches are scarce.

We present a comparison between RISH and a PCR method for detecting and genotyping EBV infection in 41 children with B-cell NHL. We also describe PCR conditions resulting in similar sensitivity and reliability to RISH, to validate PCR as a first, rapid diagnostic method, followed by RISH for the diagnosis of EBV-associated NHL.

## Methods

### Patients and clinical samples

Forty-four children (1–15 years old), diagnosed with NHL at the Instituto Nacional de Câncer (INCa), Rio de Janeiro, Brazil, were studied. The Ethics committee of INCa approved this study.

The sample included 38 Burkitt's lymphomas/L3-ALL (BL), 2 Burkitt's-like lymphomas (BLL) and 4 diffuse large B-cell lymphomas (DLBCL). Histopathological diagnosis was revised according to the R.E.A.L classification [15]. In 41 cases, paraffin-embedded tumor tissue (PET) samples were available for RISH/PCR comparisons. In 16 cases, PET and fresh tumor samples were compared. In 9 cases, bone marrow (BM) mononuclear cells and tumor mass samples were studied simultaneously. BM infiltration was assessed by morphological and molecular procedures.

Thirty peripheral blood (PB) samples from healthy donors and 26 reactive lymph nodes from HIV-negative patients without history of previous cancer, referred to the laboratory for clonality detection, were used as controls.

### EBER-1 RNA *in situ *hybridization

EBV infection was diagnosed by RISH using riboprobes for EBER1 as described [16,17]. PET sections were deparaffinized, rehydrated, digested with proteinase K, and hybridized overnight at a concentration of 0.25 ng/μl of the biotinylated probe. Detection was accomplished with a streptavidin-alkaline phosphatase conjugate. Slides were counterstained with methyl green and mounted with resin. One case of EBV-positive Burkitt's lymphoma was used as positive control; cells expressing EBER1 showed dark nuclear staining. Analysis was performed blindly respect to PCR assays.

### PCR amplifications

High molecular weight (HMW) DNA was obtained by conventional methods [18]. PET-DNA was extracted following strict measures to avoid cross-contamination. Suitability of DNA for PCR amplifications was assessed in single and multiplex reactions for amplifying four constitutive genes, as described [19].

EBV genotyping was performed by nested-PCR [20]. The first PCR reaction amplified a common region of *EBNA2 *followed by two separate nested reactions amplifying distinctive regions (Table 1). Single-PCR assays with both type-1 and -2, specific primers were also performed. DNA was amplified in 50 μl reactions containing 1.5 mM MgCl_2_, gelatin 0.001%, 0.3 μM of each primer, 1U of *Taq Platinum DNA polymerase *(Invitrogen) and, alternatively, either 500 ng of HMW DNA, 5 μl of lysate or 1 μl of first reaction for nested-PCR. Cycling conditions: First reaction: 94°C 2 min, 35 cycles of 94°C 1 min, 52°C 90 sec, 72°C 4 min, followed by 72°C for 10 min. Nested reaction: 94°C, 2 min, 35 cycles of 94°C 30 sec, 52°C 1 min, 72°C 2 min, followed by 72°C for 10 min.

**Table 1:** Primers used for *EBNA2 *PCR typing

**Primers**	**Sequence (5'-3')**	**Use in the PCR reaction**	EBNA-2 Location*
EBNA-2F	TGGAAACCCGTCACTCTC	1^st ^reaction sense	48572-89
EBNA-2I	TAATGGCATAGGTGGAATG	1^st ^reaction sntisense	49355-73
EBNA-2C	AGGGATGCCTGGACACAAGA	Nested reaction sense	48810-29
EBNA-2G	GCCTCGGTTGTGACAGAG	Nested reaction antisense type-1	49048-65
EBNA-2B	TTGAAGAGTATGTCCTAAGG	Nested reaction antisense type-2	2020-39#

* Sequence locations for type-1 correspond to B95.8 coordinates (Accession number V01555) and for type-2 (#) to AG876 isolate (Accession number K03332).

Reproducibility was assessed through a blind PCR test with two separate DNA extracts obtained from each of 15 different PET samples. We also compared HMW-DNA and PET-DNA amplifications of 9 patients. The EBV-negative Ramos and the EBV-positive Raji and BC1 cell lines were used as negative and positive controls for type-1 and type-2 PCR assays, respectively. PCR reactions were performed at least twice, in a PTC-100TM (MJ Research. Inc., Watertown, MA) thermocycler.

Sensitivity assays were performed with DNA extracts from sequential, 10-fold dilutions of Namalwa cells containing two integrated EBV genomes per cell [21] in an EBV-negative background.

To test the possibility of EBV amplification in samples lacking morphological or molecular evidence of malignancy, BM aspirates and tumor samples from the same patients were compared in 9 cases. Presence of clonal immunoglobulin (IGH) rearrangements was investigated using consensus primers (FR3-JH and FR2-JH) [19]. Once identified in the tumor mass, clonal markers were investigated in BM for detecting infiltration at the molecular level.

## Results

### RISH detection and comparisons with PCR assays

Infection was detected in 28 of 41 of patients (68%). In the first set of parallel assays, EBV was detected by RISH in 27/41 cases and by PCR in 28/41 cases. Discordant results were one RISH-positive/PCR-negative and two RISH-negative/PCR-positive (7%). When both assays were repeated in different samples of the same tumor mass, the RISH-positive/PCR-negative case was confirmed while one RISH-negative/PCR-positive was shown to be RISH-positive/PCR positive and the other, RISH-negative/PCR-negative. Considering RISH as the standard test for EBV detection, RISH resulted in one false-negative case (sensitivity 96%) while PCR produced one false-negative result, corresponding to 96% sensitivity, 100% specificity, 100% positive predictive value and 93% negative predictive value.

### Molecular detection

**Figure 1:**
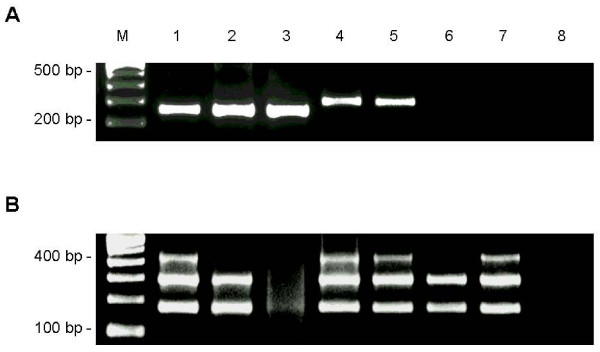
**Molecular analysis of EBV-positive and negative Non-Hodgkin lymphomas**. (A) Nested-PCR EBV genotyping. Expected sizes of nested PCR products were 250 bp (type-1) and 300 bp (type-2), amplified from an 801 bp fragment obtained in the first PCR reaction. Lane 1: Type-1 positive control (Raji cell line); lanes 2–3: EBV-positive type-1 patients; lane 4: Type-2 positive control (BC1 cell line); lane 5: EBV-positive type-2 patient; lane 6: EBV negative patient; lane 7: negative control (Ramos cell line); lane 8: PCR control (without DNA). (B) DNA amplification testing by multiplex PCR of constitutive β-globin, β-actin and *Glyceraldehide*-*3 phosphate dehydrogenase *genes (from bottom to top) corresponding to patients and controls in A. 2.5% agarose gel stained with ethidium bromide. M: molecular weight marker (100 bp ladder).

Amplifications of constitutive genes from PET-DNA were successful in 38/41 samples (93%). Single and nested-PCR assays for amplifying the *EBNA2 *gene were performed in all samples (fig 1). Infection was detected in 28 of 41 (68%) cases. Analysis of EBV amplifications in the PET-DNA samples showed three PCR-positive/RISH-positive cases in which constitutive genes could not be amplified by multi- or singleplex. In these samples, high levels of DNA degradation did not seem to impair viral DNA amplification since, in all cases, the 801 bp, first step- and the 250/300 bp single- PCR products were amplified. A comparison of RISH and PCR results is shown in Table 2, which includes PCR results of cases with constitutive amplification.

**Table 2:** Comparison of EBV detection by RISH and PCR and EBV typing in children with NHL

	EBV Detection		EBV Typing	
	
Diagnosis	EBER-ISH+ (N = 41)	PCR+ (N = 38*)	Type-1 (% of infected)	Type-2 (% of infected)
BL	25/35	21/32	18	3
BLL	0/2	0/2	0	0
DLCL	3/4	4/4	2	2
Total	28 (68%)	25 (66%)	20 (80%)	5 (20%)

* Only cases with amplification of cellular genes were used for calculation of frequencies

Nested and single PCRs, as well as blind PCR tests, with two separate DNA extracts from 15 PET samples, were completely concordant, as were comparisons of HMW-DNA and PET-DNA amplifications.

**Figure 2:**
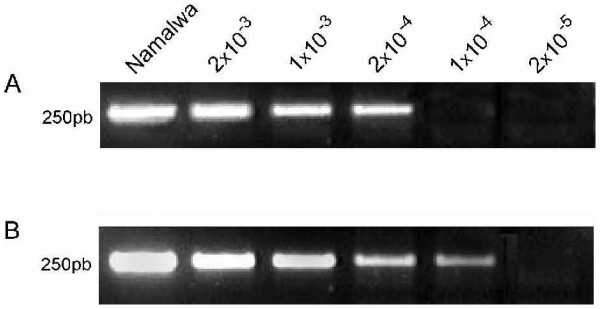
**Sensitivity assays**. Namalwa cells (2 EBV genome per cell) were serially diluted in the EBV-negative cells of Ramos cell line. PCR results showed that the method is able to detect 1 EBV genome in a background of 5 × 10^3 ^(first reaction) (A) and 1 × 10^4 ^negative cells (nested PCR) (B). 2.5% agarose gel stained with ethidium bromide.

EBV-DNA could be detected at a minimal dilution of 1 Namalwa cell in 2 × 10^4 ^EBV-negative cells by single PCR, with a 0.5 log_10 _increase by nested PCR (fig 2).

Presence of EBV was investigated in HMW-DNA from 30 healthy PB and 26 polyclonal lymphoproliferations. None of the PB and one reactive lymph node (3.8%) showed positive results by single and nested EBV-PCR assays. In the 9 BM aspirates from BL patients, the EBV genome was detected only in BM samples infiltrated by EBV-positive tumor cells (Table 3).

**Table 3:** PCR EBV detection in bone marrow of NHL patients according to infiltration status in RISH- and PCR -positive and negative cases

**Patient N°**	**Morphology BM**	Clonality* BM	**EBER-ISH Tumour**	**EBV-PCR Tumour**	**EBV-PCR BM**
19	Infiltrated	+	+	+	+
28	Infiltrated	-†	N	N	N
34	Not Infiltrated	N	N	N	N
36	Not Infiltrated	N	+	+	N
37	Not Infiltrated	+	+	+	+
38	Not Infiltrated	N	+	+	N
42	Not Infiltrated	N	+	+	N
43	Not Infiltrated	N	ND	N	N
44	Not Infiltrated	N	ND	N	N

* Monoclonal markers were identified by FR3/JH and FR2/JH amplifications, sensitivity 1–2 × 10^-3^; † case lacking clonal marker; BM, bone marrow; N, negative; ND, not done.

## Discussion

One of the most striking characteristics of pediatric BL is the variant frequency of EBV association in different geographic regions [5]. In tropical Africa it is almost always EBV-related. Conversely, in sporadic BL in developed countries, EBV association has been demonstrated in 15 to 30% of cases [5,22]. The association of BL and EBV in developing countries is intermediate between the sporadic and endemic types [8,23,24]. In South America, a high association of EBV and BL was reported in the Northeast of Brazil (~80%) [25-27], and a lower association was observed in patients from Argentina and Chile [28-30]. In the present study, we detected a frequency of EBV association of 68% in 41 childhood NHL from Southeastern Brazil, which is higher than in developed countries. Thus, the use of EBV for identifying new therapy targets in poor-risk, EBV-positive lymphomas is of interest, leading to an effort to improve current EBV diagnosis.

EBER-RNA *in situ *hybridization is the standard for EBV diagnosis in tumor cells [14] while PCR procedures are used for EBV typing. Although the simplicity of PCR might favor its adoption as a first-line method for diagnosis, its high sensitivity may produce false positive results due to detection of EBV-positive memory cells and/or non-tumor, bystander lymphocytes. However, the frequency of EBV-positive memory cells in healthy seropositive individuals accounts for less than 1/50,000 in almost all estimates [3,4,31] while BL and DLBCL are mainly characterized by lymphoproliferation of monomorphic cells carrying a high viral load when infected by EBV [31]. These observations prompted us to validate PCR assays for EBV diagnosis in these high-grade NHL, where strictly standardized PCR methods may have an important role. First, in a high complexity center offering cancer care to a large population, RISH is a second or third timeline method, considered only after histopathological and immunohistochemical diagnosis. The early definition of EBV status is important in some situations, for instance, to define the enrolment of a patient in a clinical protocol or in a viral load monitoring study, or to proceed with biological studies on fresh material. Second, the financial cost of RISH may be limiting in low-resource countries, making the effort to develop a rationale for EBV diagnosis, including PCR as a first-line, rapid approach followed by RISH for confirmation, significant.

We present a specific and reliable method for EBV-detection and typing in clinical samples. The choice of PET-DNA as PCR template aimed to make the results of both methods comparable, and to test the reliability of PCR in the most unfavorable technical conditions. Our comparisons of EBV detection by RISH and PCR showed that both methods provided highly concordant data (95%) with the same sensitivity (96%). The low EBV detection in reactive lymphoproliferations (4%) also points to the suitability of adopting PCR as a routine screening test, which can be instrumented based on single round PCR, decreasing the risks of potential cross-contamination.

Experiments of nested-PCR assays with Namalwa well-preserved DNA showed the highest sensitivity of our method. Even this sensitivity excluded the possibility of detecting EBV-bearing memory cells in clinical samples, reinforced by paired analyses of 9 BM and tumor samples, in which EBV was detected by PCR only in cases showing BM infiltration by EBV-positive tumor cells. It should be mentioned that the proposed diagnosis scheme depends on the sensitivity of our PCR method, because a more sensitive method might lead to a different conclusion.

In previous PCR typing studies in BL, concordant RISH/PCR results were reported in RISH-positive cases that were subsequently PCR-tested [23-30]. As no information was provided on PCR results in EBER-negative cases, an exceedingly high sensitivity of those EBV-specific PCR methods, making them unsuitable for diagnostic purposes, could not be assessed.

In this study, although only one viral gene was tested, viral DNA appeared to be more efficiently amplified than PET genomic DNA, raising the possibility that viral DNA might be more resistant to degradation, probably due to a differential effect of the tissue fixative on EBV DNA/protein complexes [32]. However, if PCR is used as a screening test, amplification of cellular genes should be considered mandatory.

The molecular detection of EBV in lymphomas has not produced coherent results due to biological heterogeneity and to methodological differences [33-35]. The diagnostic criteria recommended by the IARC-WHO [14] are appropriate for entities like HD, where the heterogeneity of clinical subtypes and cell types justify this conservative approach [35]. Molecular EBV detection should also be cautiously considered in peripheral T-cell NHL and anaplastic large-cell lymphoma, since they comprise heterogeneous entities with uncertain clonality status [36,37].

## Conclusion

The high correlation we observed between RISH and PCR data suggests that EBV diagnosis could be updated for lymphomas like BL and DLBCL, with PCR as a rapid approach followed by RISH confirmation. This would be adequate for EBV-diagnosis in developing regions, especially in areas where the epidemiological status of several EBV-associated entities remains unclear [26,27].

## Competing interests

The author(s) declare that they have no competing interests.

## Authors' contributions

RH designed the study, performed and interpreted molecular and RISH analyses and wrote the manuscript. LRW, CGS and FEF performed and interpreted molecular analyses. DEO performed and interpreted RISH analyses. CEK revised clinical-pathological data of cases. CEB interpreted RISH data and revised critically the manuscript. HRS revised critically and participated in manuscript elaboration. IRZ designed the study and revised critically the manuscript. All authors read and approved the final manuscript.
